# What Keeps a Man off the Panel?

**Published:** 1914-05-16

**Authors:** Thomas Johnston

**Affiliations:** 91 Carlisle Road, Hove


*To the Editor of* The Hospital.

### What Keeps a Man Off the Panel?

*To the Editor of* The Hospital.

Sir,—Dr. Brock may be, as he says, a "scientific" psychologist, but he is not logical in his letter of May 3. Is it "lack" of faith in himself that keeps a man off the panel?

I venture to suggest that a non-panel man has considerable faith in himself and in his profession. He is able to throw himself more keenly into his work than he could do under the restrictions of the panel, and it is by that means he secures the greater confidence of the public.

There are some who would sit on the wall. If a list could be published of men who are not on the panel, the public would find that list useful. The men who sit on the wall might find it of some value to be on that list. Some who are now on the panel would perhaps find it more remunerative to come off. Some distinction must be made.—I remain, yours faithfully,

Thomas Johnston.

91 Carlisle Road, Hove, May 11, 1914.

